# Combination ATR-FTIR with Multiple Classification Algorithms for Authentication of the Four Medicinal Plants from *Curcuma* L. in Rhizomes and Tuberous Roots

**DOI:** 10.3390/s25010050

**Published:** 2024-12-25

**Authors:** Qiuyi Wen, Wenlong Wei, Yun Li, Dan Chen, Jianqing Zhang, Zhenwei Li, De-an Guo

**Affiliations:** 1 School of Pharmacy, Guangdong Pharmaceutical University, Guangzhou 510006, China; 2112140309@stu.gdpu.edu.cn; 2 Zhongshan Institute for Drug Discovery, Shanghai Institute of Materia Medica, Chinese Academy of Sciences, Zhongshan 528400, China; weiwenlong@simm.ac.cn (W.W.); liyun2@simm.ac.cn (Y.L.); zhangjianqing@simm.ac.cn (J.Z.); lizhenwei@zidd.cn (Z.L.); 3 School of Chinese Materia Medica, Nanjing University of Chinese Medicine, Nanjing 210023, China; chendan@simm.ac.cn

**Keywords:** Curcumae Radix, classification algorithms, ATR-FTIR, species identification

## Abstract

Curcumae Longae Rhizoma (CLRh), Curcumae Radix (CRa), and Curcumae Rhizoma (CRh), derived from the different medicinal parts of the *Curcuma* species, are blood-activating analgesics commonly used for promoting blood circulation and relieving pain. Due to their certain similarities in chemical composition and pharmacological effects, these three herbs exhibit a high risk associated with mixing and indiscriminate use. The diverse methods used for distinguishing the medicinal origins are complex, time-consuming, and limited to intraspecific differentiation, which are not suitable for rapid and systematic identification. We developed a rapid analysis method for identification of affinis and different medicinal materials using attenuated total reflection-Fourier-transform infrared spectroscopy (ATR-FTIR) combined with machine learning algorithms. The original spectroscopic data were pretreated using derivatives, standard normal variate (SNV), multiplicative scatter correction (MSC), and smoothing (S) methods. Among them, 1D + MSC + 13S emerged as the best pretreatment method. Then, t-distributed stochastic neighbor embedding (t-SNE) was applied to visualize the results, and seven kinds of classification models were constructed. The results showed that support vector machine (SVM) modeling was superior to other models and the accuracy of validation and prediction was preferable, with a modeling time of 127.76 s. The established method could be employed to rapidly and effectively distinguish the different origins and parts of *Curcuma* species and thus provides a technique for rapid quality evaluation of affinis species.

## 1. Introduction

Traditional Chinese medicine (TCM) has been served as the primary natural reservoir for preventing and treating diseases for thousands of years. It has evolved a unique medicinal theoretical system through prolonged practice, thereby making remarkable contributions to human health and wellbeing [1,2]. Nevertheless, the complex chemical composition, variations in geographical origins, and differences in species origins leads to significant challenges for quality control and threat, thereby challenging the efficacy and safety of TCM in clinical practice [3]. Accurate identification of species serves as the fundamental premise for rational use of TCM. Therefore, employing modern analytical methods to delve deeper into the species origin and chemical profiles of TCM plays a crucial role in its quality assessment and control.

Fourier transform infrared spectroscopy (FT-IR) obtains chemical fingerprint spectra of samples by monitoring the spectral absorption of molecular vibrations, enabling high-throughput, rapid, non-destructive, and cost-effective analysis. Particularly when used in conjunction with an attenuated total reflection (ATR) attachment [4], it becomes an effective and powerful analytical tool for the quality evaluation of traditional Chinese medicine (TCM) [5,6,7,8]. Chemometrics has gradually become the preferred method for analyzing complex systems due to its powerful data processing capabilities. For ATR-FTIR spectra, appropriate data preprocessing methods could eliminate spectral interference noise, system variations, and other irrelevant signal influences, laying the foundation for subsequent multivariate statistical analysis. Various types of classification algorithms have been applied to chemometric analysis of fingerprint spectra. For example, ATR-FTIR spectra combined with four classification algorithms were used to distinguish six animal furs; the results showed that LS-SVM and PLS-DA could yield the best results, both achieving 100% accuracy during rapid identification.

*Curcuma* species are perennial herbaceous plants in the Zingiberaceae family, mainly distributed in Southeast Asia and with a widespread distribution in southern China, comprising approximately 10 species [9]. Among them, Curcumae Radix (CRa) and Curcumae Rhizoma (CRh), which originated from different parts in Curcuma species, could be used as the typical representative of multi-origin herbal medicine with excellent clinical application. According to Chinese Pharmacopoeia (2020 edition), the dried tubers of *Curcuma* species, including *C. kwangsiensis* (S. G. Lee et C. F. Liang), *C. wenyujin* (Y. H. Chen et C. Ling), *C. phaeocaulis* Val., *C. Longa* L.), are collectively listed as the original plants of CRa (CKRa, CWRa, CPRa, and CLRa, respectively), While the rhizomes are listed separately as the original plants of Curcumae Rhizoma (CKRh, CWRh, and CPRh) and Curcumae Longae Rhizoma (CLRh), resulting in the phenomenon of original plant overlap but differentiation among the three TCM [10]. The terpenoids and curcuminoids are primary active ingredients in *Curcuma* species, which possess a variety of pharmacodynamic effects (such as anticancer, anti-inflammatory, and neuroprotection) [11,12]. In clinical applications, adulteration is one of the most serious issues, and is difficult to distinguish among the different origins and similar substances, thereby necessitating the development of identification methods with strong specificity and high accuracy [13]. Literature reports indicate that inter-species identification of the four *Curcuma* species is often conducted using thin-layer chromatography [14], spectral technologies [15], chromatographic technologies [16], and chromatography-mass spectrometry technologies [17]. Although these detection techniques are capable of identifying species with accuracy, reliability, and high sensitivity, they also face challenges such as intricate sample preparation, being time consuming, and requiring high technical proficiency [18]. Hence, there is considerable research value in exploring rapid, operationally easy, and non-destructive detection methods as alternatives for identification of affinis species.

In this study, ATR-FTIR combined with seven classification algorithms (Optimized Trees, Optimized Discriminants Analysis, Optimized SVM, Optimized KNN, Optimized Naive Bayes, Optimized Ensemble, and Optimized Neural Network) was used to rapidly identify the dried rhizomes and tubers of four *Curcuma* species. Eight kinds of medicinal materials (CKRa, CWRa, CPRa, CLRa, CKRh, CWRh, CPRh, and CLRh) were collected and the data were acquired using ATR-FTIR. Multiple spectral pretreatment methods (such as normalization, derivatives, standard normal variate (SNV), multiplicative scatter correction (MSC), and smoothing (S)) were used to process the raw data of ATR-FTIR spectral from four *Curcuma* species. After optimal pretreatment screening, seven machine learning algorithms were utilized to construct the best *Curcuma* species classification models, followed by validation and testing. Thus, a high-throughput, non-destructive, rapid, and accurate method was developed for detection and identification of affinis TCM, which provided new insights into the identification and differentiation of multi-origin herbal medicines.

## 2. Experimental

### 2.1. Sample Collection

**Figure 1:**
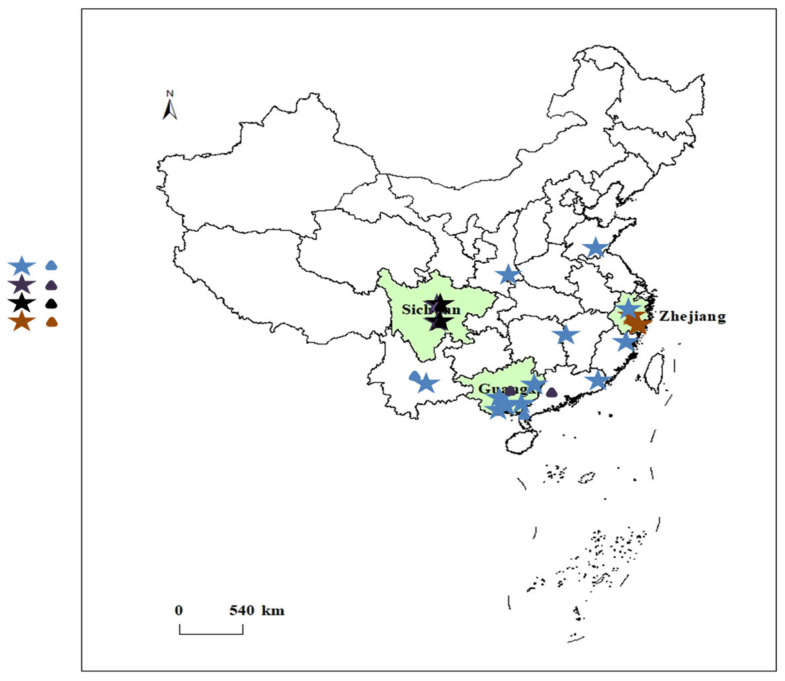
Main producing areas of eight kinds of medicinal materials.

Samples of different parts (rhizomes, tuberous roots) of four *Curcuma* species were collected between July 2022 and November 2023, yielding a total of 133 samples. The corresponding numbers and detailed sample information are presented in Table 1 and Figure 1. Among them, CKRh (*n* = 7), CKRa (*n* = 26), CWRh (*n* = 16), CWRa (*n* = 16), CPRh (*n* = 15), CPRa (*n* = 14), CLRh (*n* = 31), and CLRa (*n* = 8) were identified by botanist Shuai Yao as dried rhizomes and tuberous roots of *C. kwangsiensis*, *C. wenyujin*, *C. phaeocaulis*, and *C. longa*. In addition, 43 batches of Curcuma species of unknown origin were collected from the market.

### 2.2. Instruments and Software

Instruments included the Agilent Cary 630 Fourier transform infrared spectrometer (Agilent Technologies Co., Ltd., Palo Alto, CA, USA), desktop vacuum dryer (Shanghai Yiheng Scientific Instrument Co., Ltd., Shanghai, China), multifunctional grinder (Yongkang Hongtaiyang Mechanical and Electrical Co., Ltd., Yongkang, Zhejiang, China), and 100-mesh stainless steel sieve (Shaoxing Shayu Huafeng Hardware Instrument Co., Ltd., Shaoxing, Zhejiang, China), and Agilent 1260 Infinity II (Agilent Technologies Co., Ltd., Palo Alto, CA, USA). Software used were Origin 2022 (OriginLab Corporation, Northampton, MA, USA), SIMCA 14.1 (Umetrics AB, Umea, Sweden), and MATLAB 2022b (MathWorks Inc., San Diego, CA, USA).

### 2.3. FTIR Spectral Acquisition Experiment

FTIR spectra were collected using the Agilent Cary 630 equipped with an ATR accessory and Agilent MicroLab software. The spectral scanning range was 4000–400 cm^−1^, with 64 scans per signal accumulation and a resolution of 4 cm^−1^. After sample collection, samples were dried, crushed, sieved through a 100-mesh sieve, and stored in 2.0 mL EP tubes at 4 °C. Before collecting spectra, the ATR sampling platform was cleaned, and blank background spectra were obtained. A small spoonful of the sample was then placed on the sampling platform, covering the ATR crystal completely for spectra collection. All measurements were made in triplicate.

### 2.4. Data Analysis

#### 2.4.1. Spectral Data Preprocessing

Chemometrics is often combined with spectral data preprocessing for multivariate statistical analysis to eliminate impurity signals, which is considered a crucial step in the chemometric modeling process [19,20]. To eliminate the negative effect of different pretreatment methods on the prediction accuracy of the model, multiple pretreatment methods can be used in a complementary way to eliminate the influence left by using only one method [21].

The data from individual samples were consolidated, followed by vertical axis normalization and correction for different days. SIMCA was employed to compare preprocessing techniques, including first-order and second-order derivatives (1D/2D), SNV, MSC, and different point smoothing models (9, 11, 13, and 15). The Kennard–Stone (K-S) algorithm [22] was applied to divide the average spectral data into training (100 samples) and prediction sets (33 samples) in a ratio of 3:1. Based on Bayesian optimization, a classification learner was utilized to encompass spectral variations for all samples measured spectra and conduct 5-fold cross-validation to avoid overfitting [23].

In order to adjust the spectral intensity of all samples to a similar level, normalization was used to scale spectra and reduce the impact of sample distribution and particle size variations, which facilitated subsequent data pretreatment [24]. Smoothing, a widely useful method, can eliminate random noise and retain useful information by spectral filters [25]. Based on derivative algorithms, derivatization can subtract background and baseline drift from spectra, with 1D capable of eliminating constant baseline drift and 2D addressing slope removal [26]. Additionally, SNV and MSC are employed to deal with instrumental errors like low signal intensity or scattering [27].

#### 2.4.2. T-Distributed Stochastic Neighbor Embedding (t-SNE)

An exploratory analysis is generally used to reveal the overall outline of the sample, scout the data structure, and determine whether there are trends in the dataset. To visualize the distribution trend of the samples, a non-liner unsupervised learning method, t-SNE, was used to extract or transform the characteristics information based on a manifold learning strategy [28]. It was employed to reduce the high-dimensional data to two dimensions and output them into a visualization tool for 2D visualization with several parameters that need to be tuned for optimization, including the algorithm, distance, NumPCAComponents, perplexity per, and LearnRate.

#### 2.4.3. Classification Algorithms

In chemometrics, classification in machine learning is extensively used to analyze massive data, extract valuable information, and build models to offer objective characterization and differentiation [29].

In the discrimination of rhizomes and tubers of *Curcuma* species, the K-S method is used for dataset partitioning based on Euclidean distance, which has proved to be superior to random selection alone [23,30]. Classification trees, simple and easy interpretable models, calculate the distance between the unknown samples and known nearest neighbors of different eigenvalues for classification [31]. k-Nearest neighbor (KNN) is a commonly used non-parametric classification method that calculates the distance between the unknown samples and known nearest neighbors in the feature/attribute space to achieve classification [32]. Notably, the class size of samples and the random spectral noise are the significant factors in the classification results of KNN [24].

Discriminant analysis (DA) is one of the commonly used discriminative dimensionality reduction and classification methods, used to predict the group membership of a newly sampled observation [33,34], which has been widely studied and applied [35].

Support vector machine (SVM) is a machine learning method based on artificial intelligence technology and is used to discover the best hyperplane that maximizes the margin between different class data points. Thus, it can successfully deal with regression problems, pattern recognition, and classification and has been applied to the identification of Chinese herbal medicines [36], the prediction of chemical properties [37], and the study of structure-activity relationships of chemical components of Chinese medicines [38].

Ensemble learning (EL) constructs and combines several individual machine learning algorithms to mine data features and improve generalization performance [39]. Specifically, it extracts a set of features with a variety of transformations to produce weak predictive results by multiple learning algorithms to reduce the potential for overfitting of the training data. Therefore, it can handle small sample sizes better and fuse results with various voting mechanisms to achieve better performances than that obtained from any constituent algorithm alone [40].

Naive Bayes classification (NB) is a simple probabilistic classification algorithm based on Bayes’ theorem with the assumption of independence between characteristic attributes. The algorithm is designed to train on a given dataset for predictive performance by predicting function [41,42]. However, detecting the connection between all random variables, which is a combinatorial optimization task, is time-consuming [43].

The neural network model (NN), a complex nonlinear dynamic system, is an advanced machine learning algorithm typically used in classification or regression applications, which has strong adaptability, learning ability, fault tolerance, and robustness. As such, so it can effectively deal with nonlinear problems and has been well used in numerical prediction [44,45].

### 2.5. Chromatographic Conditions and Method Validation

#### 2.5.1. Sample Preparation

The final sample preparation conditions were as follows: 0.5 g of dried sample powder was ultrasonically (37 kHz, 1100 w) extracted with 20 mL 70% methanol at room temperature for 45 min, followed by centrifugation at 14,000 rpm for 10 min. Subsequently, the supernatant was filtered with a 0.22 μm membrane filter and injected into the HPLC instrument.

#### 2.5.2. Chromatographic Conditions

To validate the ATR-FTIR results, HPLC analysis was performed on an Agilent 1260 Infinity IIHPLC system coupled with a diode array detector (Agilent, Santa Clara, CA, USA). The chromatographic separation was performed on Diamonsil C18(2) (4.6 mm × 250 mm, 5 mm) at a flow rate of 0.8 mL/min at 25 °C. The gradient program of the mobile phases consisted of 0.1% (*v*/*v*) phosphoric acid (A) and acetonitrile (B), and was set as follows: 0–5 min, 48% B; 5–18 min, 48%–53% B; 18–35 min, 53%–75% B; 35–50 min, 75%–95% B; 50–65 min, 95% B. The injection volume was 10 μL. The detector was monitored at 210 nm and 422 nm.

The specificity, precision, stability, repeatability, and accuracy were applied to validate the developed HPLC assay method following the national standards.

#### 2.5.3. Method Validation

In accordance with the above steps “2.3”, ATR-FTIR spectral data of 43 batches of commercially available samples were collected and pre-processed. Based on the constructed identification model in the study, it was exported to the workspace. Subsequently, the infrared data were imported into the model and run to obtain the prediction results of commercially available samples. Finally, the results were verified with the HPLC fingerprints of the samples to determine the final accuracy of the model predictions.

## 3. Results and Discussions

### 3.1. Interpretation of ATR-FTIR Spectrum Features

**Figure 2:**
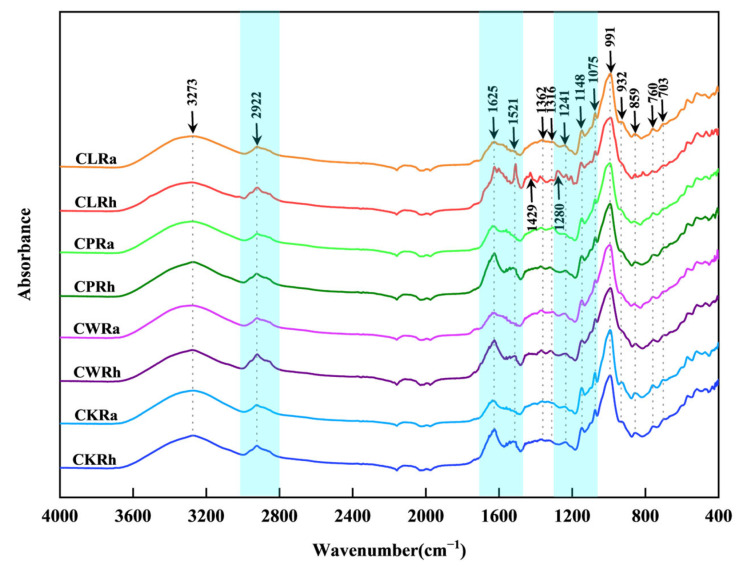
The average spectra of rhizomes and tubers of *Curcuma* species.

The average ATR-FTIR spectra of eight TCMs after vertical axis normalization and correction are shown in Figure 2. It can be seen that there are extremely overlapped spectral characteristics. The ATR-FTIR spectra of rhizomes and tubers of different *Curcuma* species exhibit significant vibrational bands near 3273 cm^−1^, 2922 cm^−1^, 1625 cm^−1^, 1362 cm^−1^, 1240 cm^−1^, 1148 cm^−1^, 1075 cm^−1^, 991 cm^−1^, 930 cm^−1^, 857 cm^−1^, 760 cm^−1^, and 703 cm^−1^. Peaks near 3273 cm^−1^ correspond to broad and intense O-H stretching vibrations. The absorption peak (distributed near 2922 cm^−1^) is primarily caused by -CH3 and-CH2 symmetric and asymmetric stretching vibration. The absorption peak distributed at 1625 cm^−1^ is attributed to stretching vibration of carbonyl (C=O) conjugated to other double bonds, C=C stretching vibrations, and aromatic stretching vibration [46,47]. For the absorbance at 1362 cm^−1^, methyl and methylene C-H bending vibrations are observed. The spectral bands near the 1316–1050 cm^−1^ range represent the aromatic ring (=C-H) inner-plane bending and stretching vibrations of C-C or C-O bonds [47,48]. The absorbance at 991 cm^−1^ is mainly assigned to stretching vibration of C-OH and -CH3 rocking vibrations and aromatic ring (=C-H) inner-plane bending [47,48]. The infrared spectrum, which peaks at 930–700 cm^−1^, originates from aromatic skeleton vibrations (due to out-of-plane bending vibrations of C-H) [46].

Notably, around 2922 cm^−1^ and 1522 cm^−1^, there are significant C-H stretching vibrations and the vibrational mode of C=C double bonds or ring breathing in the rhizomes of *Curcuma* species, which can be used to differentiate them from tubers. Furthermore, differences between the spectra of CLRh and the other seven herbs in the range of 1534~1183 cm^−1^ are more pronounced, possibly due to higher content of curcuminoid components in Curcuma [46,49].

These results indicate certain differences among the rhizomes and tubers of the four *Curcuma* species. However, due to the significant overlap, rapid identification of these four *Curcuma* species from ATR-FTIR spectra is challenging. Therefore, chemometric analysis of spectral data is necessary to achieve rapid identification.

### 3.2. Exploratory Analysis by t-SNE

Due to the holistic and complex nature of fingerprint spectra, it is necessary to observe the differences in the chemical space distribution of samples through unsupervised exploratory analysis. As depicted in Section 3.1, the overall infrared spectral profiles of plants exhibit high similarity, making manual differentiation challenging. Therefore, visual analysis methods are required to reduce high-dimensional spectral information to an observable level. Typically, studies utilize unsupervised PCA and supervised PLS-DA analyses to visualize scatter plots of principal components and latent variables for data exploration. However, these linear analyses often require extraction of 10 or more principal components and latent variables to effectively extract differential information from the original data, and there are widespread complex nonlinear relationships among variables, resulting in unsatisfactory predictive performance [50]. Recently, t-SNE has attracted increasing attention [28], and was initially employed to visualize the results in this study.

**Figure 3:**
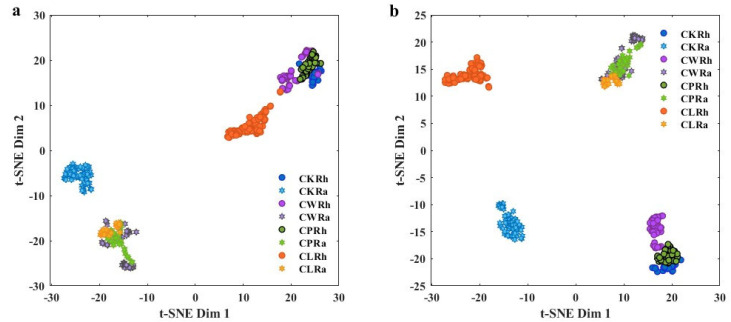
t-SNE visual analysis results of rhizomes and tubers of four *Curcuma* species, (**a**) raw spectra, (**b**) pre-processed spectra.

In practice, the parameters for the optimal t-SNE analysis are as follows: Algorithm: exact, Distance: cosine, NumPCAComponents: 20, Perplexity: 40, LearnRate: 2000. The matrix of raw (pretreatment) ATR-FTIR spectra data were composed of 399 rows (sample) and 1924 columns (1920 variables). As illustrated in Figure 3, each point represents a sample, different colors represent different origins Curcuma species, and if the two samples are similar, the points will be clustered together owing to their equal attributes. In Figure 3a, all samples are divided into three clusters, with CRh and CLRh located in the top right corner and CRa situated in the bottom left corner, where CKRa samples are grouped together and clearly distinguished. This distance shows that the difference between rhizomes and tuberous roots is more obvious than the interspecific differences, and CKRa can be well distinguished from other species. After 1D + MSC + 13S pretreatment (Figure 3b), the clustering effect of the same color points is enhanced, which means some of the interference factors are eliminated to display better interspecies differences. In terms of distance distribution, the difference between CLRh and CRh and different origins of CRh is more evident. However, multi origins of CRa and CRh still cluster together individually, with CWRa being the most dispersed, making precise species differentiation challenging based on overall fingerprint profiles. Therefore, subsequent attempts would be made to use supervised pattern recognition methods to accurately differentiate the rhizomes and tubers of four *Curcuma* species.

### 3.3. Selection of the Best Pretreatment Methods

In general, when multivariate data are obtained from FTIR for qualitative and quantitative analysis, pretreatment methods are applied to correct for scattering, baselines changes, peak shifts, noises, missing values, and other effects of systematic errors [51]. In this study, 1D/2D, MSC, and SNV were applied to preprocess the acquired spectra. Based on the SVM model, the accuracy of the model training set and the test set under the raw data and 16 preprocessing methods were compared. As shown in Table 2, the raw data have accuracy around 99.7% and 98% for the classification error of the incorrect division of CWRa. To acquire better differentiation, a series of pretreatment portfolio strategies were trained and predicted. As can be seen from the accuracy, 1D had the excellent classification ability for this dataset, obtaining an accuracy of nearly 100% for the training set, while the accuracy of 2D is lower than the raw data. The results indicate that improper selection of pretreatment methods may negatively affect the accuracy and interpretability of the model [52]. Moreover, it is found that the accuracy of the training set and the test set in the 1D + SNV + 9S, 1D + MSC + 13S, and 1D + SNV + 15S modes are all 100%, which displays the advantages that distinguish from other portfolio strategies of pretreatment in the terms of accuracy and speed. The training time of 1D + MSC + 13S is the shortest and the repetition effect is better between the three models. Finally, 1D + MSC + 13S can demonstrate good differentiation ability, which is regarded as an alternative method for the data preprocessing of the classification of *Curcuma* species.

### 3.4. Comparison of Different Model Classification Algorithms

**Figure 4:**
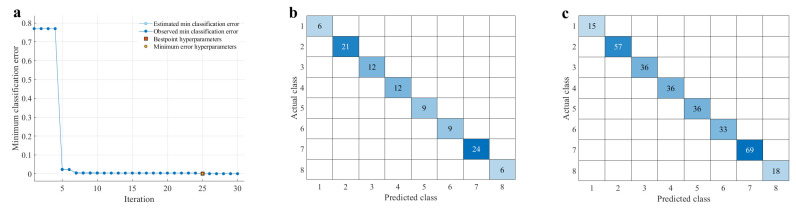
The performances of SVM models based on 1D + MSC + 13S spectral data, (**a**) minimum classification error, (**b**) confusion matrices of testing set, (**c**) confusion matrices of training set.

We sought to illustrate the potential of ATR-FTIR combined with classification algorithms in the identification of eight types of Chinese medicine. The comparison among different model classification algorithms ought to determine which one is more suitable to develop an efficient classification model [24]. Combined with the best result of pretreatment methods, seven useful classification algorithms, including optimizable trees, DA, SVM, KNN, NB, EL, and NN, were established for comparison using a default Bayesian optimizer. This technique is particularly suited for optimization of high-cost functions, situations where the balance between exploration and exploitation is important [53]. Accuracy and training time of datasets were used to evaluate the classification ability of each pattern recognition method. The minimum classification error and confusion matrices of different classification models established for the detection of each species can be found in Figure S1 (Supporting Information). The classification results of each algorithm for different datasets were compared and are displayed in Table 3 and Figure 4.

The prediction results of the training set showed that the optimal trees model took the shortest time with 114.07 s, but the accuracy was 98.7%. While the optimal NB model took the longest time and had an accuracy of 99.7%. DA, SVM, and NN obtain a similar accuracy (100.0%), which imply the established methods could implement *Curcuma* species classification correctly based on the 1D + MSC + 13S. Although the obtained results are satisfactory to a certain extent, it made an apparent difference when the three methods were utilized to predict the class of test samples. In the optimal DA and NN (shown in Figure S1C,G (Supporting Information)), 3 of the 12 samples of CWRa were misclassified as CLRa. In addition, one batch of CPRa was misclassified as CLRa in the NN model, which suggested that it was not applicable. This indicated that there was a great challenge in the classification of CWRa. Intuitively, SVM performed better for the discrimination of *Curcuma* species, which showed that all the corresponding number labels of Chinese medicinal herb were correctly classified with 100% accuracy in 127.76 s. From Figure 4a, it could be concluded that the best point hyperparameters and the minimum error hyperparameters were confirmed when the iteration of the SVM model was 25. The optimal results are obtained when using multi-class methods: one to all, box constraint level: 915.3893, kernel function: gaussian. To avoid deviations in the calculation results, the data was used 10 times. The accuracy of the method could reach 100%, which indicated that the method had good repeatability based on the data matrix of the calibration set (100 × 3 samples × 1290 variables).

### 3.5. Validation of SVM Model with HPLC Fingerprints

To verify the reliability of the SVM model in predicted classification results, this study used commercially available samples of unknown *Curcuma* species for species identification. At the same time, the HPLC fingerprint of the confirmed original *Curcuma* species was used to identify the varieties of the commercial samples so as to judge the accuracy of the model results.

As shown in Table 4, it contained the predicted class labels of the SVM model and the identification species of the HPLC fingerprint based on the 43 batches of commercially available samples. It revealed that the results of the model were highly consistent with the chromatographic identification results, which meant the constructed discrimination model is reliable. Moreover, the commercially available samples included seven kinds of Curcuma species, all except CKRh, and most of the samples collected were 2 (CKRa) and 8 (CLRh), which were speculated to have a wide liquidity.

Combined with literature research, ATR-FTIR combined with SVM models shows prominent characteristics in terms of high throughput, fast speed, and convenient performance compared to conventional data analysis (as shown in Table 5).

## 4. Conclusions

In the study, a high-throughput, rapid, and effective method was established for identification of different origins and parts of *Curcuma* species based on ATR-FTIR spectrum and classification algorithms. First, vertical axis normalization and correction for various time intervals were performed on the ATR-FTIR spectral data. Second, sixteen combinations of derivatives, MSC, SNV, and smoothing point numbers were compared to select the optimal preprocessing method. 1D + MSC + 13S, as an established pretreatment method based on SVM model, was more effective and applicable to pre-treat the FTIR spectroscopic data. Models were constructed by classifying the preprocessed data through t-SNE analysis of raw and preprocessed data, as well as seven classification algorithms (Trees, DA, Optimized SVM, Optimized KNN, Optimized NB, Optimized EL, and Optimized NN). The results indicate that the constructed SVM model exhibited the best classification ability, correctly identifying both the training and test sets with an accuracy of 100% after preprocessing with 1D + MSC + 13S. Compared to the other six models, the SVM model demonstrates higher accuracy and shorter runtime with the advantages of simplicity, speed, and accurate classification. Meanwhile, the SVM model also had the drawback of being unable to correctly recognize those materials belonging of mixed samples and other medicinal materials, resulting in classification errors. In future work, we will concentrate on exploring how to utilize characteristic attribute better to design for non-target species removal, thereby further improving the algorithm’s performance. In summary, ATR-FTIR combined with various classification models could serve as a rapid and effective method for identifying different parts of four Curcuma species, providing new insights into the identification and differentiation of multi-origin herbal medicine.

## Figures and Tables

**Table 1:** Basic information of eight kinds of medicinal materials.

NO.	Samples	Botanical Origins	Name	Area	Number	Collection Time
1	CKRh	*C. kwangsiensis*	Curcumae Rhizoma	Guangxi	7	2023.02–2023.09
2	CKRa		Curcumae Radix	Guangxi	18	2023.02–2023.11
				Others	8	2023.07
3	CWRh	*C. wenyujin*	Curcumae Rhizoma	Zhejiang	16	2023.01–2023.11
4	CWRa		Curcumae Radix	Zhejiang	16	2023.01–2023.09
5	CPRh	*C. phaeocaulis*	Curcumae Rhizoma	Sichuan	15	2023.03–2023.11
6	CPRa		Curcumae Radix	Sichuan	14	2023.03–2023.09
7	CLRh	*C. longa*	Curcumae Longae Rhizoma	Sichuan	31	2022.07–2023.07
8	CLRa		Curcumae Radix	Sichuan	8	2023.03–2023.09

**Table 2:** Pretreatment results of Curcuma species with different modes.

Modes	Training Set Acc (%)	Test Set Acc (%)	Prediction Speed (obs/s)	Training Time (s)
Raw	99.7	98	720	167.69
1D + MSC + 9S	99.7	96	540	227.78
1D + MSC + 11S	100	96	580	180.39
1D + MSC + 13S	100	100	690	127.76
1D + MSC + 15S	100	97	530	197.84
2D + MSC + 9S	98.7	96	450	178.84
2D + MSC + 11S	99.3	94.9	400	200.31
2D + MSC + 13S	99.3	94.9	320	346.8
2D + MSC + 15S	99.7	100	700	144.64
1D + SNV + 9S	100	100	690	354.8
1D + SNV + 11S	100	97	570	363.04
1D + SNV + 13S	100	97	530	413.46
1D + SNV + 15S	100	100	730	399.28
2D + SNV + 9S	97.7	97	400	538.38
2D + SNV + 11S	98.7	97	380	440.78
2D + SNV + 13S	99.7	100	700	205.46
2D + SNV + 15S	99.3	100	660	457.67

**Table 3:** Classification results of *Curcuma* species with different classifiers.

Models	Training Set Acc (%)	Test Set Acc (%)	Prediction Speed (obs/s)	Training Time (s)
Trees	98.7	98	370	114.07
DA	100	97	360	156.66
KNN	99	90.9	230	164.44
SVM	100	100	690	127.76
NB	99.7	97	14	3629.5
EL	99.3	100	460	380.3
NN	100	96	610	1470.4

**Table 4:** The results of the model prediction and HPLC fingerprint identification of 43 batches of commercially available samples.

Samp	Predicted Class Label	HPLC	Samp	Predicted Class Label	HPLC	Samp	Predicted Class Label	HPLC
S-1	3	3	S-19	7	7	S-36	2	2
S-2	3	3	S-20	7	7	S-37	2	2
S-3	3	3	S-21	7	7	S-38	2	2
S-4	4	4	S-22	7	7	S-39	2	2
S-5	4	4	S-23	7	7	S-40	2	2
S-6	4	4	S-24	8	8	S-41	2	2
S-7	4	4	S-25	8	8	S-42	2	2
S-8	5	5	S-26	8	8	S-43	2	2
S-9	5	5	S-27	7	7	Acc		100%
S-10	5	5	S-27	7	7			
S-11	6	6	S-28	5	5			
S-12	6	6	S-29	7	7			
S-13	6	6	S-30	7	7			
S-14	7	7	S-31	7	7			
S-15	7	7	S-32	7	7			
S-16	7	7	S-33	2	2			
S-17	7	7	S-34	2	2			
S-18	7	7	S-35	2	2			

**Table 5:** Comparison of SVM model with traditional methods.

	Chromatography	Traditional NIR/ATR-FTIR	1D + MSC + 13S and SVM
Advantages	Can separate target compounds, wide suitability, high resolution, selectivity, sensitivity, and fully automatable operation; the related active substances or quality control markers screening	Rapid, applicable to both raw materials and processed samples, less consumption (chemical solvents and reagents), non-destructive, environmentally friendly, enables online analysis	Rapid, high accuracy, non-destructive and non-invasive, better tolerance, environmentally friendly, minimum sample preparation and more convenient
Disadvantages	Time-consuming (for sample preparation, injection, and data processing), standardize operations, high cost for reagents and sophisticated instruments	Signal overlapping, no separation capacity, needs to be supported by chemometrics methods	For other medicinal materials, another model needs to be built according to the process, otherwise the output will be incorrect
Reference	[54,55]	[15,56,57]	

## Data Availability

Data are contained within the article.

## Supplementary Materials

The following supporting information can be downloaded at: https://www.mdpi.com/article/10.3390/s25010050/s1, Figure S1: Minimum classification error and confusion matrix for different classification models (A–H) (a. Minimum classification error, b. confusion matrix for testing set, c. confusion matrix for training set). Figure S2: HPLC fingerprints of 43 batches of commercially available samples.
