# Supplementary material for: Combination ATR-FTIR with Multiple Classification Algorithms for Authentication of the Four Medicinal Plants from Curcuma L. in Rhizomes and Tuberous Roots
Source: Sensors (Basel). 2024 Dec 25;25(1):50. doi: 10.3390/s25010050 (PMC11722871; doi:10.3390/s25010050)
Supplement: Supplementary file 1 [file sensors-25-00050-s001.zip › sensors-3323396-supplementary.pdf]

Supplementary Materials

Combination ATR-FTIR with Multiple Classification Algorithms for Authentication of the Four Medicinal Plants from *Curcuma* L. in Rhizomes and Tuberous Roots

Qiuyi Wen [1,2,†], Wenlong Wei [2,†], Yun Li [2], Dan Chen [3], Jianqing Zhang [2], Zhenwei Li [2] and De-an Guo [1,2,*]

[1] School of Pharmacy, Guangdong Pharmaceutical University, Guangzhou 510006, China; 2112140309@stu.gdpu.edu.cn
[2] Zhongshan Institute for Drug Discovery, Shanghai Institute of Materia Medica, Chinese Academy of Sciences, Zhongshan 528400, China; weiwenlong@simm.ac.cn (W.W.); liyun2@simm.ac.cn (Y.L.); zhangjianqing@simm.ac.cn (J.Z.); lizhenwei@zidd.cn (Z.L.)
[3] School of Chinese Materia Medica, Nanjing University of Chinese Medicine, Nanjing 210023, China; chendan@simm.ac.cn
* Correspondence: daguo@simm.ac.cn; Tel.: +86-21-20271516; Fax: +86-21-50272789
† These authors contributed equally to this work.

Comparison of different model classification algorithms

To illustrate the potential of ATR-FTIR combined with seven classification algorithms in the identification of eight types of Chinese medicine, the comparison between different analytical techniques, different chemometrics methods are needed to determine which one is more appropriate to develop an efficient strategy. The minimum classification error and confusion matrices of different classification models established for the detection of each species can be shown in Fig. S1(A-G). Firstly, we analyze (c) the classification results of the training set, followed by (b), and finally combined with the model's (a) minimum classification error.

Fig. S1(A) shows the training set of the raw data has misclassified 4 (CWRa) as 6 (CPRa). Processed by different classification algorithms, in the optimal DA, NN and SVM, shown in Fig. S1(C, G, H), obtain a similar accuracy (100.0%) of training sets(c), which means the proposed methods can realize *Curcuma* species classification. Further analyzing the testing sets(b), we can find that in the optimal DA and NN, 3 of the 12 samples of CWRa were misclassified as CLRa. In addition, one batch of CPRa was misclassified as CLRa in the NN model, which suggested that it was not applicable. Comparison of the results of the confusion matrices for the different datasets revealed that 4 (CWRa) is the most likely to be misclassified as other species among the seven models. In contrast to all models, the SVM model exhibits excellent classification capabilities (see Fig. S1(H)) with all eight kinds of TCM herbs can be correctly categorized. From (c) the minimum classification error, it can be seen that the optimal parameters are obtained at the 25th iteration. The optimal results are obtained when using multi-class methods: one to all, box constraint level: 915.3893, kernel function: gaussian.

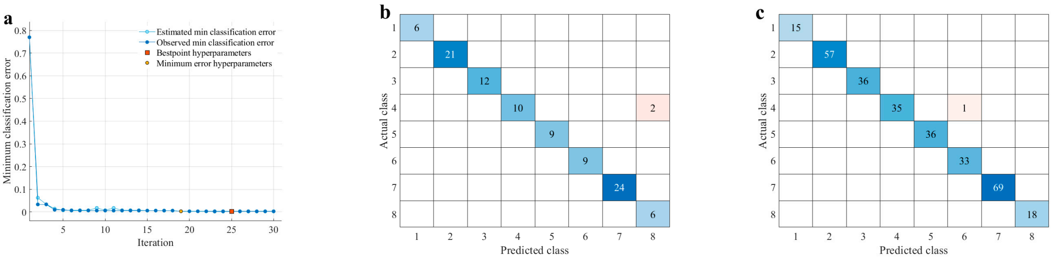

(A) Raw Data

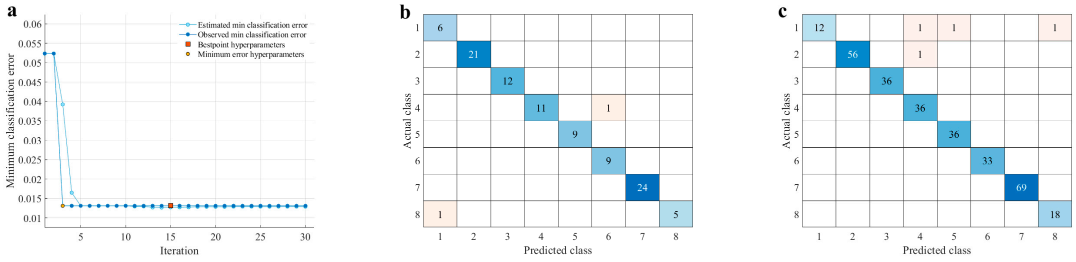

(B) Decision Trees

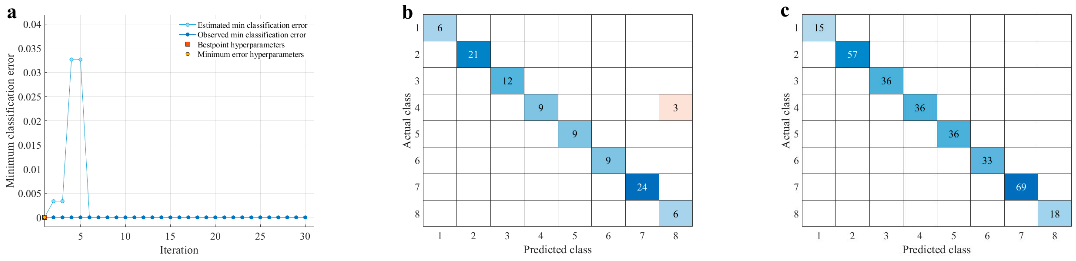

(C) Discriminant Analysis

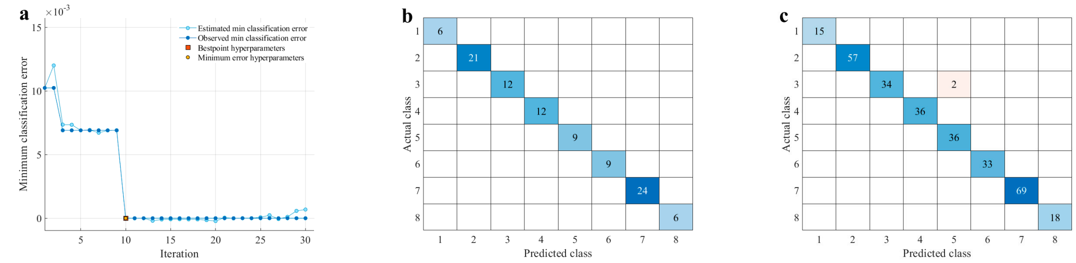

(D) Ensemble learning

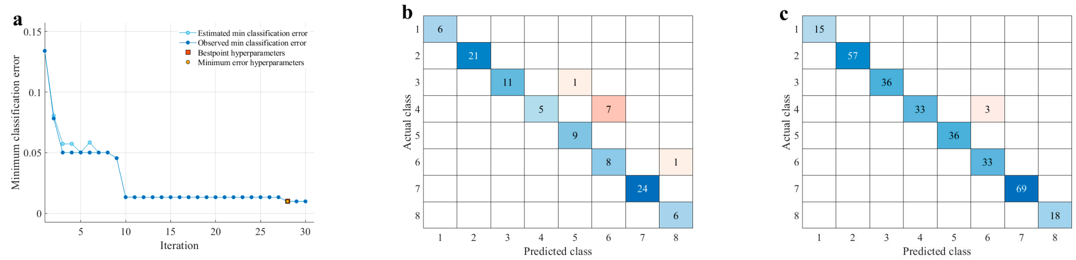

(E) K Nearest Neighbors

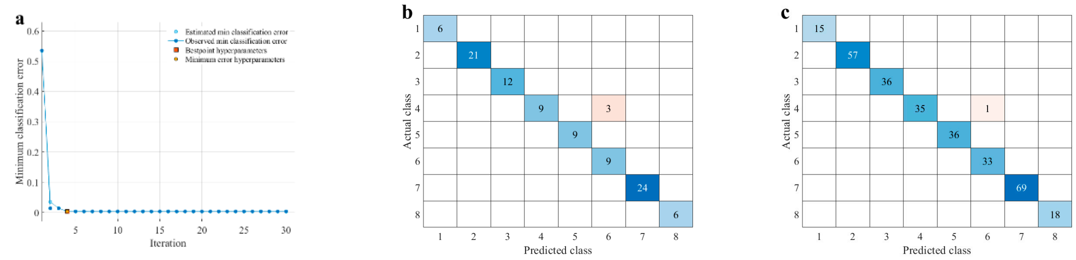

(F) Naive Bayes classification

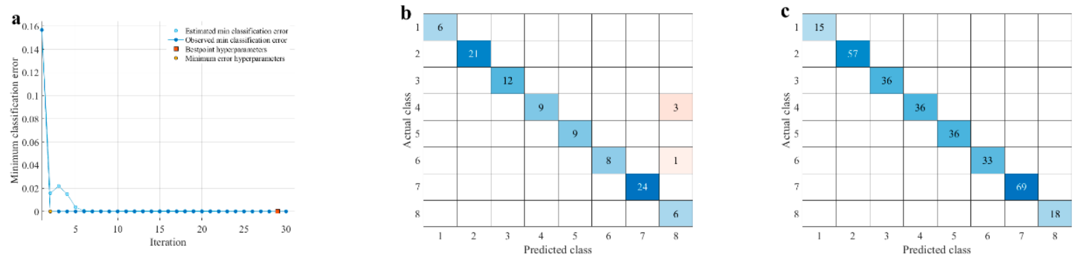

(G) Neural Network

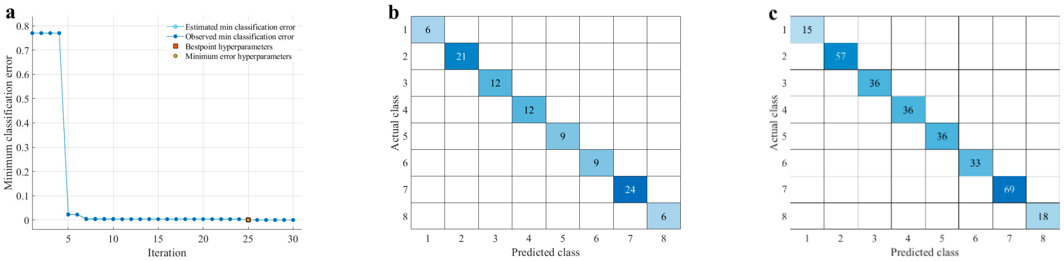

(H) Support Vector Machine

Figure S1. Minimum classification error and confusion matrix for different classification models (**A-H**) (a. Minimum classification error, b. confusion matrix for testing set, c. confusion matrix for training set).

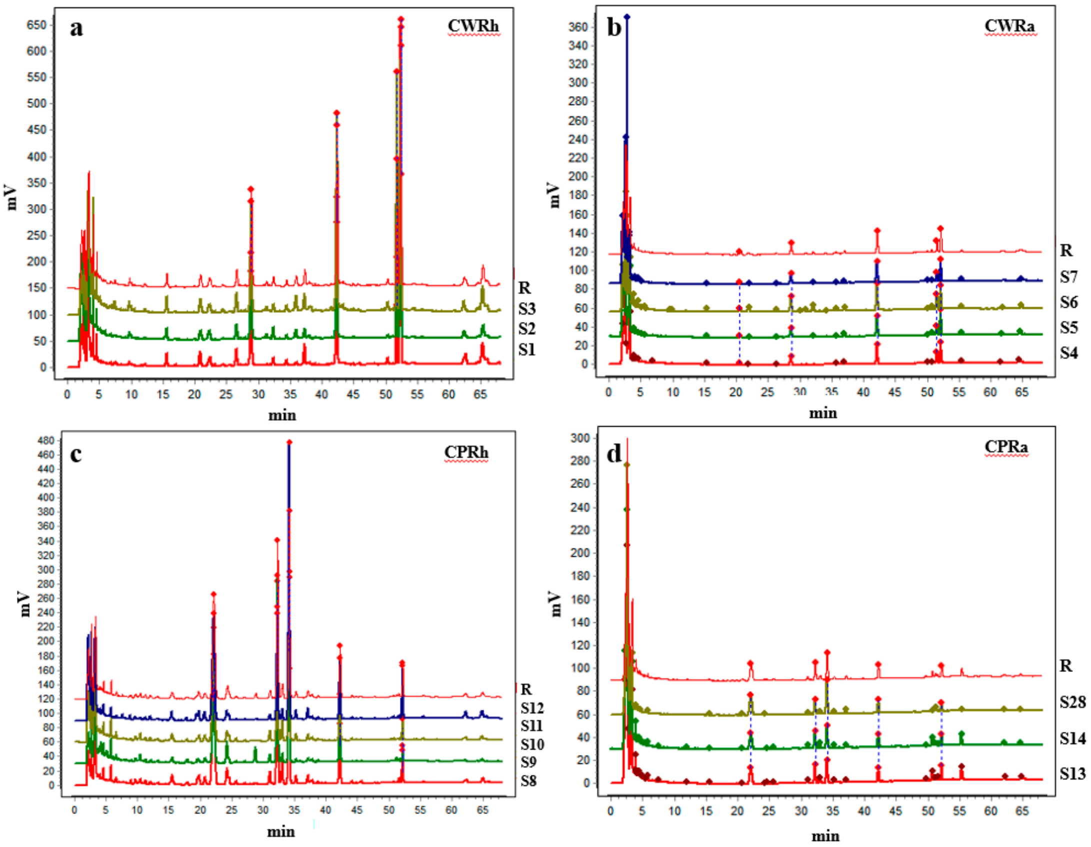

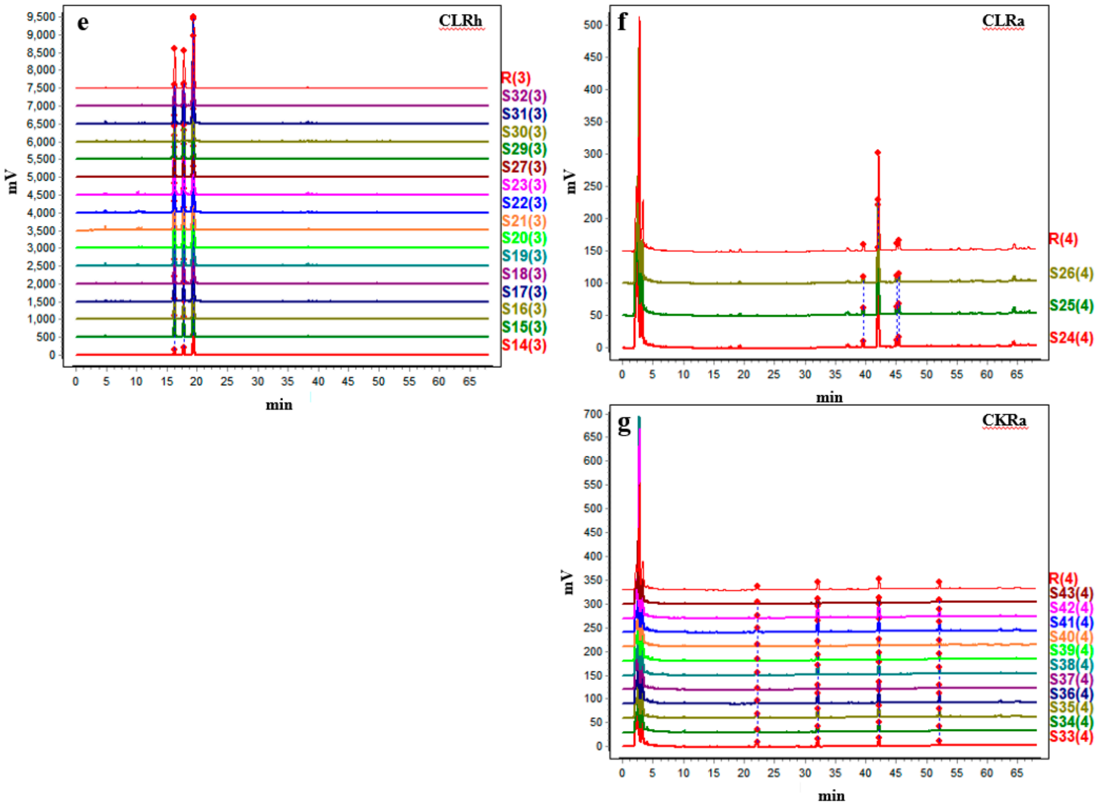

Figure S2. HPLC fingerprints of 43 batches of commercially available samples.
